# Field efficacy of fluralaner (Bravecto^®^ chewable tablets) for preventing *Babesia canis* infection transmitted by *Dermacentor reticulatus* ticks to dogs

**DOI:** 10.1186/s13071-023-05820-2

**Published:** 2023-07-27

**Authors:** Rafael Chiummo, Eva Zschiesche, Balázs Capári, Róbert Farkas, Mathieu Chiquet, Dhimitër Rapti, Rezart Postoli, Alain Audry, Michael Leschnik

**Affiliations:** 1 MSD Animal Health Innovation GmbH, Zur Propstei, 55270 Schwabenheim, Germany; 2 Kapriol Bt., Sümeg, 8330 Hungary; 3 Department of Parasitology and Zoology, University of Veterinary Medicine, Budapest, 1078 Hungary; 4 Artemis Animal Health, 7, rue du Clos Rouillé, Champtocé-sur-Loire, France; 5 Faculty of Veterinary Medicine, Agricultural University of Tirana, Tirana, Albania; 6 Clinique Vétérinaire, 2c Route de Grayan, 33780 Soulac sur Mer, France; 7 University Clinic for Small Animals, Veterinary University Vienna, Vienna, Austria

**Keywords:** *Babesia canis*, Babesiosis, *Dermacentor reticulatus*, Dog, Fluralaner

## Abstract

**Background:**

The isoxazoline fluralaner is effective for prevention of *Babesia canis* transmission from infected *Dermacentor reticulatus* ticks to dogs for 84 days in a controlled environment. This study was designed to evaluate the effectiveness of fluralaner chewable tablets for sustained prevention of *B. canis* infection of dogs in endemic areas under natural conditions.

**Methods:**

In Europe, privately owned, clinically healthy pet dogs were enrolled and randomized either to receive fluralaner at 25–56 mg/kg (Bravecto^®^ chewable tablets) on days 0 and 84, or to remain untreated during the *D. reticulatus* season. Blood samples were collected to evaluate *B. canis* exposure: on days 0 and 21 (exposure before day 0), during the study and at the end of the tick season (dogs suspected of having become infected after day 0). Efficacy was determined by the percentage reduction in *B. canis* transmission risk based on the difference in *B. canis*-positive tests in fluralaner-treated dogs compared with untreated dogs. In addition, ticks collected at monthly intervals throughout the study were identified to species level and females tested for *B. canis* DNA.

**Results:**

A total of 152 dogs were enrolled in the study, although nine dogs were excluded because they tested positive for *B. canis* DNA or antibodies within 21 days after enrollment. During the study period, no fluralaner-treated dog became positive for *B. canis*, resulting in calculated efficacy of 100%. However, babesiosis infection was diagnosed in five untreated control dogs (Fisher’s exact test, left-sided, *P* = 0.0312). Tick analyses revealed that one sample collected in Hungary was infected with *B. canis*.

**Conclusion:**

Oral administration of Bravecto chewable tablets at the recommended dosage to dogs completely prevented *B. canis* transmission under field conditions in an endemic area for 12 weeks.

**Graphical Abstract:**

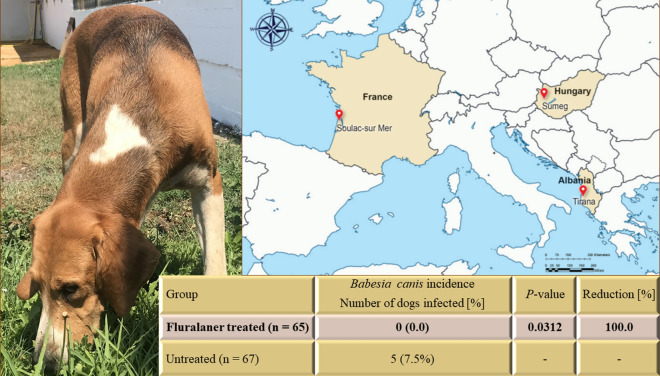

## Background

Infections with tick-transmitted *Babesia* spp. are increasingly diagnosed in both animals and people [1–5]. In dogs, infections can remain asymptomatic or cause multi-organ failure, with a risk of death. The wide range of clinical signs include apathy, weakness, anorexia, pale mucous membranes, fever, enlarged lymph nodes and spleen, thrombocytopenia, jaundice, pigmenturia and anaemia that is caused by a combination of intravascular and extravascular hemolysis resulting from parasite-caused rupture of red blood cells and the activity of secondary immune-mediated processes [1, 6].

In Europe, reported infections with *Babesia canis* align with the expanding geographical distribution of its primary vector, the ornate dog tick *Dermacentor reticulatus* [4–7]. Tick distribution is not homogeneous and varies between countries and between regions in each country since it is largely dependent on the habitat [8]. In 2014, the estimated annual incidence of clinical babesiosis in the Western European dog population was 0.70% (95% confidence interval 0.69–0.71%). In Central Europe, canine babesiosis-associated mortality can be as high as 20%, whereas reports in the southwest of Europe, particularly Italy, France, Spain and Portugal, may be < 5%. These differences may result from genetic diversity and variable virulence of different *B. canis* strains [9].

Transmission of *B. canis* from a tick to a susceptible host is believed to occur around 48 h following attachment [10, 11]. Therefore, infection can be prevented if an acaricide is used that causes tick paralysis and death within 24 h of the onset of tick feeding. The isoxazoline compound fluralaner has demonstrated this rapid onset of efficacy and, by providing this efficacy against *D. reticulatus* for 12 weeks, prevented *B. canis* transmission by infected ticks throughout an 84-day period under a severe laboratory challenge [12, 13]. To validate these laboratory findings under field conditions, a study was designed to evaluate the effectiveness of oral fluralaner for preventing *B. canis* transmission to dogs. This negatively controlled, randomized, examiner-masked and multicenter study was conducted in areas known to be endemic for *B. canis* during the autumn/winter tick season when adult *D. reticulatus* are most active [4].

## Methods

**Fig. 1:**
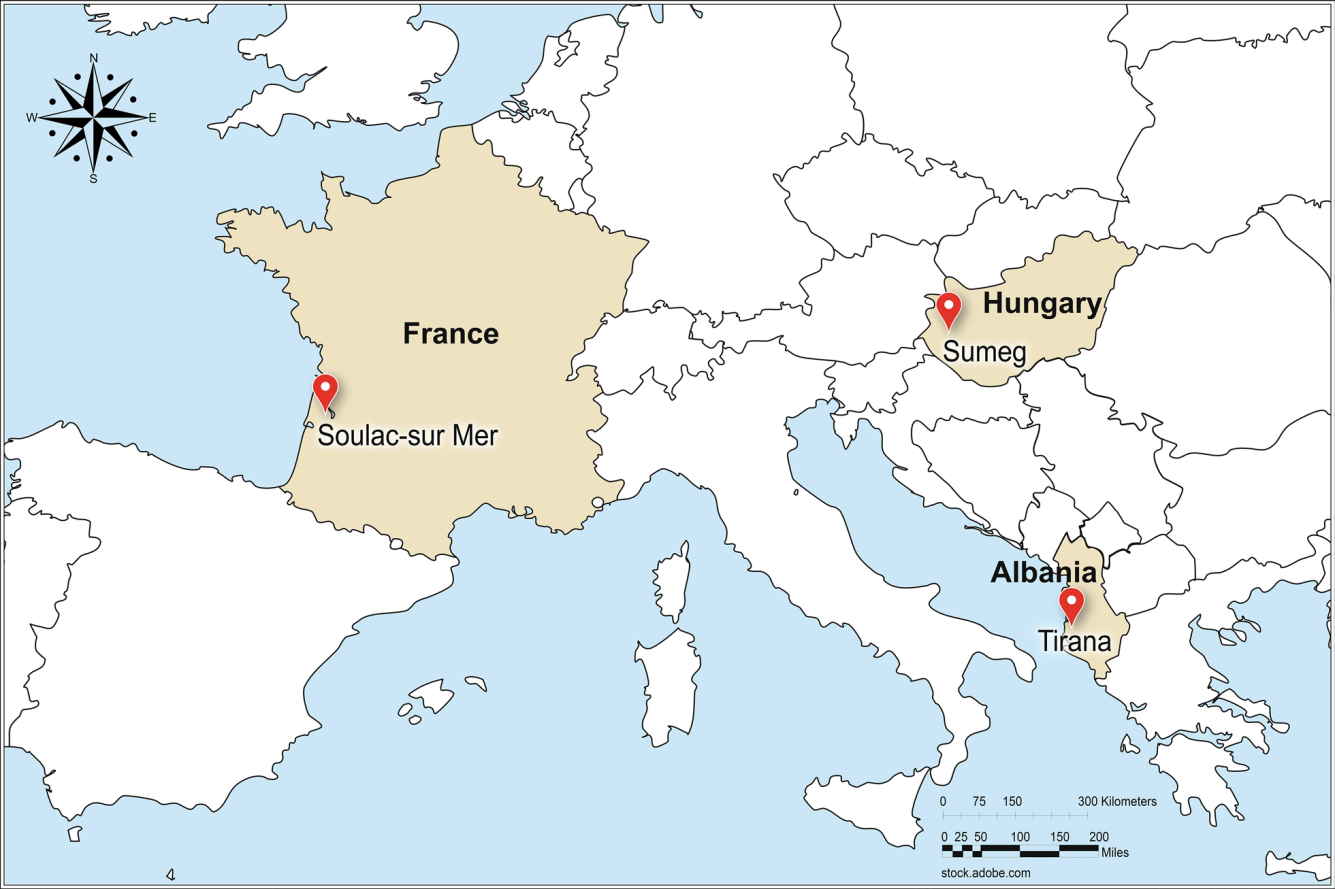
Map of the study area (prepared using Adobe Creative Cloud software, by designer Joseph Caputo)

The field study was conducted in three countries: Albania (Tirana), France (Soulac-sur Mer) and Hungary (Sumeg) (Fig. 1). Study procedures adhered to those described in Good Clinical Practices, VICH (International Cooperation on Harmonisation of Technical Requirements for Registration of Veterinary Medicinal Products) Guideline 9 [14], Statistical principles for clinical trials for veterinary medicinal products (pharmaceuticals) [15], Guideline for the Demonstration of Efficacy of Ectoparasiticides [16], and draft Guideline for Vector-Borne Diseases [17]. Sites were selected based on previous reports by local veterinarians of tick infestation and canine babesiosis transmission. Enrolled pet dogs were privately owned and clinically healthy, ≥ 8 weeks old, and weighed ≥ 2 kg. Dogs had access to outdoor areas, including regular walks in tick infested areas. Dogs were excluded for the following: pregnant or lactating; treated with acaricides or protozoicides within the labeled duration of those products prior to study enrollment; vaccinated against babesiosis; or already *B. canis*-infected.

Routine health procedures (e.g. vaccination) and medical care were continued during the study. However, owners were instructed to not use any acaricides, protozoicides or *Babesia* vaccines on their dog during the course of the study, and to keep the dog on its usual management food and water supply. Each owner provided a signed informed consent form.

Enrolled dogs were randomly allocated to study groups using computer-generated randomization lists. All dogs in one group were treated on days 0 and 84 with fluralaner (Bravecto^®^ chewable tablets for dogs, MSD Animal Health) at the label dose (25–56 mg/kg), at or around the time of feeding [18]. Dogs were weighed on a calibrated scale prior to treatment, and the chewable tablets were selected according to the recommended body weight band. Dogs in the other group were not treated with acaricides and served as negative control. Animals were kept in their normal housing conditions at the time of treatment, and two dogs from one household could be in different study groups. Grooming and bathing were not restricted during the study.

It was determined that approximately 55 dogs per study group would be required in at least two different geographical locations to provide a representative picture of the exposed dog population, allow a meaningful statistical comparison of incidence rates and minimize the number of untreated control animals due to animal welfare. With an estimated drop-out rate of approximately 9%, targeted enrollment was 60 dogs per group divided across three different countries, and no country was permitted to enroll more than 40% of the total study population.

Allowing for a maximum of 21 days following infection for *Babesia* to become detectable, blood samples were collected on days 0 and 21 and tested for pre-existing *B. canis* infections, and any positive dogs were excluded [19, 20]. Additionally, throughout the study period, blood samples were taken from any dogs clinically suspected of being infected with *B. canis* and from all dogs 21 days after the end of tick season (determined by the complete absence of ticks on study dogs) in order to detect any evidence of *B. canis* infection. Blood samples were evaluated using two different methods: detection of *B. canis* antibodies in serum (commercial enzyme-linked immunoassay [ELISA] kit, Afosa GmbH, Blankenfelde-Mahlow, Germany) and polymerase chain reaction (PCR). PCR analysis was conducted on DNA extracted from blood samples (QIAamp DNA Mini Kit, Qiagen Hilden, Germany), and later the primers BJ1 5′–GTC TTG TAA TTG GAA TGA TGG–3′ and BN2 5′–TAG TTT ATG GTT AGG ACT ACG–3′ amplified a ~ 500-base-pair fragment of the 18S ribosomal RNA (rRNA) gene [21] for pathogen identification over a 1.5% agarose gel stained with ethidium bromide and visualized under ultraviolet light. Samples were analyzed at the Department of Parasitology and Zoology, University of Veterinary Medicine, Budapest, Hungary. The positive PCR products were purified and sequenced at the Hungarian Academy of Sciences, Biological Research Centre, Szeged, Hungary, and the sequences compared to the National Center for Biotechnology Information (NCBI) Nucleotide database.

All study assessments were completed by examiners masked to treatment group. Dogs were observed daily by their owners for abnormal clinical signs, and if any were observed, an examiner was informed immediately to visit, perform a complete physical exam and take a blood sample. Any dog testing positive for *B. canis* infection was removed from the study and administered rescue treatment.

Study areas were flagged monthly, and collected ticks were stored in a labeled tube containing 80% ethanol for later species, gender and life stage identification at the University of Veterinary Medicine in Budapest, Hungary. Each specimen was identified at species level based on its morphology [22, 23]. The same PCR method described previously for blood analysis was used on pooled female ticks (maximum 10 ticks per sample) collected at each site and time point to detect *B. canis*.

Treatment efficacy was determined from the percentage reduction of the risk of *B. canis* transmission based on the incidence rate in each treatment group:

$$ {\text{Efficacy}}\,\,\left[ \% \right] = \,\frac{{{\text{Control incidence rate}} {-}{\text{Treated incidence rate}}}}{{\text{Control incidence rate}}}\, \times \,100 $$

where the incidence rate in each study group was the number of *B. canis*-infected dogs in relation to the total number of dogs. Treatment efficacy was considered to have been achieved if the percentage reduction of transmission risk was ≥ 90% [17].

The superiority of fluralaner treatment compared to the untreated control was investigated using Fisher’s exact test (one-sided) with the level of significance set to *α* = 0.025.

Reduction of transmission risk was determined from the incidence density rate in the untreated control group and the incidence density rate in the fluralaner group using the equation:

$$ {\text{Risk reduction}}\,\,\left[ \% \right] = \frac{{{\text{Incidence rate }}\left( {{\text{control}}} \right) {-}\,{\text{incidence rate }}\left( {{\text{treated}}} \right)}}{{{\text{Incidence rate }}\left( {{\text{control}}} \right)}} \, \times \,100, $$

where the incidence rate in each study group was calculated on a monthly basis as follows:

$$ {\text{IDR }}\left( {\text{per 100 cases per time}} \right) = \frac{{\text{Number of infected animals}}}{{\text{Sum of months in which each animal is negative}}}\, \times \,100. $$

## Results and discussion

Study recruitment began in September 2019 with a total of 152 dogs enrolled: 52 in Hungary, 50 in Albania and 50 in France. Twenty dogs were subsequently excluded from the assessment: nine because of a positive *B. canis* antibody titer within the first 21 days (i.e. pre-study infection), three that died from dog bite wounds, seven that were lost to follow-up and one untreated control dog that had a concomitant infection manifested by lethargy, increased temperature and conjunctivitis. Of the nine dogs removed because of asymptomatic pre-study *B. canis* infection, three were from Albania, one was from France and five were from Hungary; confirming a history of canine babesiosis in these areas as informed by the local veterinarians, eight dogs were positive on day 0 tests, and one untreated dog was positive on day 21. There were no treatment-related adverse events and the study concluded in February 2020 in Hungary (day 168) and France (day 167), and in March 2020 in Albania (day 182).

Age (4 months to 13 years), body weight (4.6–51.3 kgs), and gender (approximately 65% males and 35% females) were similar between groups. The most common breed in the study was the Great Anglo-French Tricolor Hound (approximately 40% of dogs in each group), and 26.2% of dogs were described as mixed breed in the treated group and 14.9% in the control group.

All fluralaner-treated dogs remained free of *B. canis* infection throughout the study (Table 1). In October 2019, five control group dogs developed clinical signs of babesiosis, including anorexia, icterus, hemoglobinuria, increased body temperature and conjunctivitis. Each of these dogs was confirmed by PCR, based on the sequences, to be infected with *B. canis*, further confirmed by a positive antibody test, resulting in removal from the study. All affected dogs recovered clinically following treatment with imidocarb, dexamethasone and amoxicillin. All countries had *B. canis*-infected dogs prior to day 21, and all *B. canis*-positive tests after this date were from Hungary. All dogs sampled on the final day of the study were test-negative for *B. canis*.

**Table 1:** Incidence rates and reduction of *Babesia canis* transmission risk following fluralaner treatment

Country	Study group	Incidence rate [%]	*P*-value^a^	Reduction [%]
Albania	Fluralaner-treated (*n* = 23)	0 (0.0)		
	Untreated (*n* = 24)	0 (0.0)		
France	Fluralaner-treated(*n* = 22)	0 (0.0)		
	Untreated (*n* = 23)	0 (0.0)		
Hungary	Fluralaner-treated (*n* = 20)	0 (0.0)	0.0236	100.0
	Untreated (*n* = 20)	5 (25.0)	–	–
Overall	Fluralaner-treated (*n* = 65)	0 (0.0)	0.0312	100.0
	Untreated (*n* = 67)	5 (7.5%)	–	–

^a^Fisher’s exact test, left-sided

Fluralaner treatment was 100% effective at reducing the risk of *B. canis* transmission at the end of the study period, and thus above the required ≥ 90% threshold established by the guideline for vector-borne disease [17]. Across the three countries, the low *B. canis* infection rate in the untreated control group (7.5%) resulted in a calculated *P*-value (Fisher’s exact test, left-sided, *P* = 0.0312) slightly above the predetermined superiority threshold (0.025). The efficacy of 100% did not allow a meaningful calculation of confidence limits for the difference in proportions.

In Hungary, the *B. canis* incidence rate in fluralaner-treated and untreated control dogs was 0% and 25%, respectively. The calculated *P*-value for this difference (Fisher’s exact test, left-sided, *P* = 0.0236) is significant (Table 1). Overall, the incidence density rate was 0.0 for fluralaner treatment and 1.4 for the untreated group, resulting in a 100% calculated risk reduction.

*Dermacentor reticulatus* ticks were collected from all three countries and were found in Hungary throughout the entire study period, with a peak in early October and November (Table 2), coinciding with the appearance of clinical *B. canis* infection in five untreated dogs. One tested tick sample from Hungary, collected in October, was positive for *B. canis*. It is likely that all ticks had some risk of carrying *B. canis*, and study dogs faced a constant risk of transmission. In the Czech Republic, 2.8% of ticks, and in Eastern Poland 3% of *D. reticulatus* ticks tested positive for *B. canis*, an infection frequency that confirms circulation of *B. canis* through the *D. reticulatus* population [18, 19]. The pathogen challenge level will vary under the natural conditions of a field study depending on the presence and quantity of the vector and the vector-borne pathogen. Despite the known incidence of *B. canis* in the selected areas of France and Albania among local practitioners, the enrolled dogs did not contract the infection during the study.

**Table 2:** Test results for *Babesia canis* detected in *Dermacentor reticulatus*

Country	Collection date	Number of ticks	*B. canis* status
Albania	November 2019	3 Males	Not tested
	December 2019	1 Male	Not tested
France	September 2019	1 Female	Negative
	March 2020	4 Females, 2 males	Not tested
Hungary	04 October 2019	4 Females, 16 males	Negative
	10 October 2019	33 Females, 18 males	Positive
	November 2019	187 Females, 110 males	Negative
	January 2020	26 Females, 23 males	Negative
	February 2020	19 Females, 24 males	Negative

Seasonal data for *D. reticulatus* activity indicate that dog owners should be advised to protect their dogs with acaricides throughout the year [19]. The 12-week duration of acaricidal efficacy provided by fluralaner chewable tablets has been linked to improved owner compliance with acaricidal protection recommendations, effectively providing more months per year of coverage, relative to monthly administered acaricides [24]. The speed of tick kill and the duration of effect help to explain the 100% protection fluralaner provided against *B. canis* infection under the field conditions of this study.

In conclusion, the study demonstrated that *B. canis* is present in *D. reticulatus* ticks in Albania, France and Hungary, and that oral fluralaner administration results in 100% reduction of the risk of transmission of *B. canis* for 12 weeks.

## Data Availability

Data from this study are proprietary and are maintained by MSD Animal Health.
